# Severe Measles, Vitamin A Deficiency, and the Roma Community in Europe

**DOI:** 10.3201/eid1809.111701

**Published:** 2012-09

**Authors:** Clea Melenotte, Philippe Brouqui, Elisabeth Botelho-Nevers

**Affiliations:** Aix Marseille Université, Faculté de Médecine, Marseille, France

**Keywords:** measles, viruses, vitamin A deficiency, Roma community, Europe

**To the Editor:** The Roma community in Europe is a subgroup of the Romani people, whose origins are in northern India and who have been known in English-speaking countries as “gypsies.” Measles outbreaks, including severe cases, were reported in the European Roma community during 2008-2010 (*1*,*2*). We describe the potential roles of malnutrition and vitamin A deficiency as risk factors for severe measles in adults from this community.

In Europe, >25,000 cases of measles, more than half of which occurred in France, were reported during a 2011 outbreak (*3*). The exact proportion of measles cases occurring among the Roma community in France during the outbreak are not available (*2*). Measles epidemiology has changed; the disease now mainly affects children <1 year old and young adults, the latter of whom are mostly unvaccinated or have unknown vaccination status (*2*,*4*). Roma people in Europe experience some of the worst health conditions in the industrialized world: they live in overcrowded conditions and have limited access to prevention programs and to healthcare services (*2*,*5*). In such populations, deficiencies of vitamins, such as A, C, and E, have been reported (*6*). Vitamin A deficiency has been associated with severe cases of measles in children in developing countries (*7*,*8*). To date, we did not find published data associating vitamin A deficiency with severe measles among adults. We describe 6 adults from the Roma community in Marseille, France, who had measles and low levels of vitamin A; 2 of these persons had severe measles.

Case-patients 1 and 2 were men who were 21 and 25 years of age, respectively. They were admitted to North Hospital, Marseille, France, with typical signs of measles (fever, cough, and maculopapular rash). They had no medical history of serious illness, including no immunocompromising conditions, and no history of measles vaccination. For both patients, the diagnosis of measles was confirmed by the results of PCR performed on pharyngeal and urinary samples. In case-patient 1, acute meningoencephalitis developed, and he was transferred to the intensive care unit for 3 days. During his stay, the patient was found to have active viral hepatitis B. Case-patient 2 had the following signs and symptoms: abdominal pain and vomiting, severe hepatitis (serum transaminases level 10× higher than the upper reference limit; total bilirubin within reference range), and keratitis. Other causes of viral or bacterial hepatitis were ruled out by serologic testing, and the patient did not frequently drink alcohol.

Case-patients 1 and 2 had vitamin A deficiency with values of 0.31 mg/L and 0.2 mg/L, respectively, (reference range 0.5–0.8 mg/L). We measured vitamin A levels in blood samples from the next 4 consecutive hospitalized patients with measles, all of whom were from the Roma community. They did not have complications of measles, but were hospitalized for infection-control reasons. All 4 patients had low levels of vitamin A (0.16–0.34 mg/L) (Table). In case-patient 4, the blood level of retinol-binding protein was 0.026 g/L (normal range 0.02–0.05 g/L), confirming vitamin A deficiency. Vitamin A supplementation was administered intramuscularly as recommended by the World Health Organization (200,000 IU followed by a second dose the next day) to each patient (*8*). The 6 patients progressed to recovery. Low levels of vitamin A (0.36–0.46 mg/L) were also found for 2 other patients from the Roma community who did not have measles (Table).

**Table:** Characteristics of 6 measles case-patients and 2 control patients with vitamin A deficiency, Roma community, France*

Participant	Age, y/ sex	Signs and symptoms	Defining characteristics	Vitamin A level, mg/L†	Outcome	No. days in hospital
Case-patient 1	21/M	Rash, meningo-encephalitis	Positive PCR results for pharyngeal and urinary samples	0.31	Recovered	12
Case-patient 2	25/M	Rash, hepatitis, keratitis	Positive PCR result for pharyngeal and urinary samples	0.20	Recovered	4
Case-patient 3	26/M	Rash	Positive PCR result for pharyngeal sample	0.27	Recovered	2
Case-patient 4	22/M	Rash	Positive PCR result for urinary, nasal, and pharyngeal samples; Positive serologic test result	0.27	Recovered	3
Case-patient 5	15/M	Rash	Positive PCR result for nasal sample	0.16	Recovered	1
Case-patient 6	17/M	Rash, hepatitis, Koplik spots	Clinical signs	0.34	Recovered	2
Control 1	34/F	None	NA	0.46	NA	NA
Control 2	12/M	None	NA	0.36	NA	NA

*Control participants did not have measles. NA, not applicable. †Reference range 0.5–0.8 mg/L.

Serum vitamin A concentrations do not always reflect total vitamin A stores (*9*). In severe protein–calorie malnutrition, and during intercurrent infection, serum retinol levels could be artificially low in relation to a decrease in retinol-binding protein level (*9*). However, the diagnosis of vitamin A deficiency is usually supported by low levels of serum vitamin A and levels of retinol-binding protein within the reference range as described for at least 1 of the case-patients reported here.

Vitamin A deficiency affects the severity of illness and the rate of deaths associated with measles, and it is known to induce severe measles-related complications in children, delaying recovery and promoting xerophthalmia, corneal ulcer, and blindness (*7*,*8*,*10*). Acute measles precipitates vitamin A deficiency by depleting vitamin A stores and increasing its utilization, leading to more severe ocular injury (*7*,*8*). Vitamin A supplementation given to children with measles has been associated with better outcomes (*7*,*8*). Although it is too early to associate vitamin A deficiency with severe measles in adult patients, such an association is possible. Adults with low levels of vitamin A but not infected with measles could be at higher risk for more severe disease if they become infected with the virus.

We conclude that all adults who have measles should be assessed for vitamin A and retinol-binding protein levels and should be considered for vitamin A supplementation, as are children (*8*). A prospective case-control study assessing vitamin A deficiency in the Roma adult community is necessary to assess its consequences on measles outcome. Aside from preventing complications among the Roma people, improving vaccine coverage in this nomadic population is crucial for reducing measles virus circulation among the general population.
